# Cul4A Modulates Invasion and Metastasis of Lung Cancer through Regulation of ANXA10

**DOI:** 10.3390/cancers11050618

**Published:** 2019-05-02

**Authors:** Ming-Szu Hung, Yi-Chuan Chen, Paul-Yann Lin, Ya-Chin Li, Chia-Chen Hsu, Jr-Hau Lung, Liang You, Zhidong Xu, Jian-Hua Mao, David M. Jablons, Cheng-Ta Yang

**Affiliations:** 1 Division of Thoracic Oncology, Department of Pulmonary and Critical Care Medicine, Chang Gung Memorial Hospital, Chiayi branch 61363, Taiwan; c39958@yahoo.com.tw; 2 Department of Medicine, College of Medicine, Chang Gung University, Taoyuan 33302, Taiwan; 3 Department of Respiratory Care, Chang Gung University of Science and Technology, Chiayi Campus, Chiayi 61363, Taiwan; 4 Department of Emergency Medicine, Chang Gung Memorial Hospital, Chiayi branch 61363, Taiwan; giomacky@gmail.com; 5 Department of Anatomic Pathology, Dalin Tzu Chi Hospital, Buddhist Tzu Chi Medical Foundation, Chiayi 62247, Taiwan; linpauly0018@gmail.com; 6 Department of Hematology and Oncology, Chang Gung Memorial Hospital, Chiayi branch 61363, Taiwan; loofahhsu@gmail.com; 7 Department of Medical Research and Development, Chang Gung Memorial Hospital, Chiayi branch 61363, Taiwan; jrhaulung@gmail.com; 8 Thoracic Oncology Laboratory, Department of Surgery, Comprehensive Cancer Center, University of California, San Francisco, CA 94143, USA; liang.you@ucsf.edu (L.Y.); zhidong.xu@gmail.com (Z.X.); david.jablons@ucsfmedctr.org (D.M.J.); 9 Life Sciences Division, Lawrence Berkeley National Laboratory, One Cyclotron Road, Berkeley, CA 94720, USA; jhmao@lbl.gov; 10 Department of Respiratory Care, College of Medicine, Chang Gung University, Taoyuan 33302, Taiwan; yang1946@cgmh.org.tw; 11 Department of Pulmonary and Critical Care Medicine, Chang Gung Memorial Hospital, Taoyuan branch 33378, Taiwan

**Keywords:** lung cancer, Cul4A, ANXA10, metastasis

## Abstract

Cullin 4A (Cul4A) is overexpressed in a number of cancers and has been established as an oncogene. This study aimed to elucidate the role of Cul4A in lung cancer invasion and metastasis. We observed that Cul4A was overexpressed in non-small cell lung cancer (NSCLC) tissues and the overexpression of Cul4A was associated with poor prognosis after surgical resection and it also decreased the expression of the tumor suppressor protein annexin A10 (ANXA10). The knockdown of Cul4A was associated with the upregulation of ANXA10, and the forced expression of Cul4A was associated with the downregulation of ANXA10 in lung cancer cells. Further studies showed that the knockdown of Cul4A inhibited the invasion and metastasis of lung cancer cells, which was reversed by the further knockdown of ANXA10. In addition, the knockdown of Cul4A inhibited lung tumor metastasis in mouse tail vein injection xenograft models. Notably, Cul4A regulated the degradation of ANXA10 through its interaction with ANXA10 and ubiquitination in lung cancer cells. Our findings suggest that Cul4A is a prognostic marker in NSCLC patients, and Cul4A plays important roles in lung cancer invasion and metastasis through the regulation of the ANXA10 tumor suppressor.

## 1. Introduction

Cullin 4A (Cul4A) belongs to the family of evolutionally conserved cullin proteins and it forms a multifunctional ubiquitin–protein ligase E3 complex by interacting with ring finger protein (ROC1) and damaged DNA binding protein (DDB1) [1]. The protein participates in multiple vital cellular functions, including apoptosis [2], cell cycle regulation [3], genome stability [4], nuclear excision repair [5], and histone modification [6] via the ubiquitin proteasome pathway.

Cul4A is overexpressed in several cancers, including breast [7], mesothelioma [8], lung cancer [9], and liver cancers [10]. Cul4A also causes ubiquitination and proteolysis of p53 [11], NF2 [12], RASSF1A [13], p27 [3], TGFBI [14], and p21 [8,15] tumor suppressors through different mechanisms. Cul4A physically associates with MDM2 and it participates in the proteolysis of p53 [11]. NF2 is recruited to the Roc1-Cul4A-DDB1 complex for degradation by direct interaction with the WD40-containing adaptor protein VprBP [12]. RASSF1A is a direct target of the Cul4A–DDB1 complex [13]. Cul4A–DDB1 complex associates with the F-box protein Skp2 to target p27 for proteolysis [3]. Cul4A interacts with TGFBI for ubiquitination and degradation [14]. The CDK inhibitor p21 is degraded by the PCNA coupled Cul4A–DDB1^Cdt2^ pathway [15]. In transgenic mouse models, the development of lung cancer after conditional overexpression of Cul4A has also been observed [16,17]. As a result, Cul4A plays important roles in oncogenesis and it is an attractive target for cancer therapy.

Lung cancer is the leading cause of cancer death worldwide, and surgery remains the only potentially curative treatment for early stage non-small cell lung cancer (NSCLC) patients [18]. However, post-resection recurrence rates remain high, and recurrence most commonly occurs as distal metastasis with poor prognosis and survival rates [19]. As a result, elucidating the mechanism of lung cancer metastasis is critical. Ubiquitination-mediated degradation of tumor suppressors critically affects tumor metastasis. For example, the expression of metastasis suppressor 1 (MTSS1) is regulated by SCFβ-TRCP through ubiquitination and its subsequent destruction via 26S proteasome. Reduced MTSS1 expression contributes to the increased proliferation and migration of cancer cells. Depletion of cullin 1 also leads to increased expression of MTSS1 [20], while the knockdown of Cul4A is associated with the growth inhibition of lung cancer cells [14,21]. Cul4A overexpression has been observed in 50% to 87.2% of lung cancer tissues, and it has been associated with poor prognosis of lung cancer patients in previous studies [9,22]. However, the effect of Cul4A on lung cancer cells metastasis and the association of metastasis suppressors with Cul4A is rarely explored in lung cancer cells.

The downregulation of the tumor suppressor annexin A10 (ANXA10), a member of the calcium dependent lipid binding annexin proteins family, has been observed to be associated with poor prognosis and increased metastasis in various cancers [23,24,25]. In our previous unpublished study, the upregulation of ANXA10 was observed in Cul4A knockdown lung cancer cells. Since other annexin proteins, including ANXA1 [26], A2 [27], and A7 [28] have been observed to be regulated by ubiquitination, we thus postulated that ANXA10 may also be regulated by Cul4A through ubiquitination in lung cancer cells.

In this study, the association of Cul4A expression with clinical prognosis was studied in lung cancer patients after surgical resection. The association of Cul4A with metastasis and invasion of lung cancer cells was explored both in vitro and in vivo. The associations of Cul4A with the ANXA10 tumor suppressor and regulatory mechanisms were also studied.

## 2. Material and Methods

### 2.1. Cell Lines and Cell Culture

NSCLC cell lines, including A549, H157, H460, and H322 were purchased from the American Type Culture Collection (Manassas, VA, USA). Cells were grown in RPMI-1640 complete growth medium supplemented with 10% fetal calf serum, 10 units/mL penicillin, and 10 µg/mL streptomycin at 37 °C and 5% CO_2_. The H460 and A549 Cul4A and empty vector-transfected stable cells were generated by retroviral transduction of Cul4A shRNA as described previously in [14].

### 2.2. Tissues

Fresh NSCLC and adjacent normal pleural tissues were obtained from patients undergoing surgical resection of the primary tumor after obtaining the signed consent of the patient and the approval of the Institutional Review Board of Chang Gung Memorial Hospital (IRB No. 103-6934B). Formalin-fixed, paraffin-embedded tissue samples were converted into tissue microarray (TMA) blocks using an AutoTiss 1000 arrayer (Ever BioTechnology, Taipei, Taiwan). The quality of the TMA slides was confirmed by the pathologist using hematoxylin and eosin staining.

### 2.3. Immunohistochemistry (IHC)

Formalin-fixed, paraffin-embedded tissues were cut into 4-μm sections, mounted on slides, deparaffinized with xylene, and dehydrated using a gradient ethanol series. Antigen retrieval with citric acid (pH 6.0) at 97 °C for 30 min, followed by treatment with 3% hydrogen peroxide were performed. The slides were incubated overnight at 4 °C with an anti-Cul4A antibody (Abcam, Cambridge, MA, USA) or ANXA10 (GeneTex, Irvine, CA, USA). The IHC data for the specimens were assessed using the semi-quantitative immunoreactive score (IRS). The IRS was calculated by multiplying the staining intensity (0 = no staining, 1 = weak staining, 2 = moderate staining, and 3 = strong staining) by the percentage of the positively stained cells (0 = 0% of cells stained, 1 = less than 10% of cells stained, 2 = 11–50% of cells stained, 3 = 51–80% of cells stained, and 4 = more than 81% of cells stained) as described in [29].

### 2.4. Cell Migration Assay

The cell migration assay was performed using wound-closure experiments. Cells were plated in 10 cm plates and cultured to confluence, and then scraped with a p20 tip and transferred to pre-warmed fresh media. The healing of the gap was observed at the indicated time points.

### 2.5. Cell Invasion Assay

The invasion assays were performed in 24-well (6.5 mm diameter) cell-culture inserts (8.0 mm pore size, Corning, Tewksbury, MA, USA) coated with an indicator layer of growth factor reduced Matrigel (BD Transduction Laboratories, San Jose, CA, USA). The cells were plated in the upper well in 0.2% serum and then incubated with 5% FBS and 100 ng/mL fibronectin in the lower chambers. After 24 h, cells in the upper chamber were removed with a cotton swab. Cells that migrated into the lower chamber were fixed in 4% PFA and then stained with 0.5% crystal violet. Filters were photographed and the total number of cells was quantified.

### 2.6. Western Bot Analysis

Whole protein was extracted using mammalian protein extraction reagent (M-PER) from the cell lines. The proteins were digested using the Phosphatase Inhibitor Cocktail Set II (Calbiochem, San Diego, CA, USA) and complete protease inhibitor cocktails (Roche, Lewes, UK) according to the manufacturer’s protocols. The digested proteins were separated on 4–15% gradient sodium dodecyl sulfate (SDS)–polyacrylamide gels and transferred to Immobilon-P membranes (Millipore, Billerica, MA, USA). The following primary antibodies were used: Cul4A (Abcam, Cambridge, MA, USA), ANXA10 (GeneTex), and β-actin (Sigma, St. Louis, MO, USA). After incubation with indicated secondary antibodies, the membranes were washed thoroughly and an enhanced chemiluminescence (ECL) blotting analysis system (GE Healthcare Life Sciences, Piscataway, NJ, USA) was used for antigen-antibody detection. The relative intensities of protein bands were analyzed by densitometry using ImageJ 1.46r software (National Institutes of Health, Bethesda, MD, USA).

### 2.7. Semi-Quantitative Reverse Transcription-PCR (RT-PCR) Analysis

Total RNA, extracted using the RNeasy Mini Kit (QIAGEN, Hilden, Germany) from lung cancer cells according to the manufacturer’s instructions, was transcribed to cDNA using the iScript™ cDNA Synthesis Kit (Bio-Rad Laboratories, Munich, Germany). The cDNA (2 μL) was added to a total volume of 20 μL reaction mixture and analyzed on a Bio-Rad CFX96™ quantitative PCR system (Bio-Rad Laboratories). ANXA10 (forward, 5′-gtcctatgggaagcctgtca-3′, reverse, 5′-gagaacaattgcaaccagca-3′), and actin (forward, 5′-tcgtgcgtgacattaaggag-3′, reverse, 5′-ccatctcttgctcgaagtcc-3′) primers were used for the PCR. Amplification conditions were as follows: 95 °C for 5 min, 30 cycles at 95 °C for 20 s, 56 °C for 30 s, 72 °C for 30 s, 72 °C for 5 min, and then they were maintained at 4 °C.

### 2.8. Transfection with Small Interfering RNA (siRNA) and Vectors

Pre-designed and validated Cul4A (Dharmacon, Lafayette, CO, USA), ANXA10 (Santa Cruz, Santa Cruz, CA, USA), and universal negative control siRNAs were transfected (final concentration = 50 nM) in cells grown to 80% confluence on 6-well plates using an antibiotic-free media and Lipofectamine™ RNAiMAX reagent (Invitrogen, Carlsbad, CA, USA) following the manufacturer’s instructions. At 96 h after transfection, the cells were treated with gemcitabine for 72 h followed by counting for viable cells. The pCMV6-ANXA10-GFP (OriGene, Rockville, MD, USA) and empty pCDNA3 (Invitrogen, Carlsbad, CA, USA) vectors were transfected with OmniFect™ transfection reagent (TransOMIC, Huntsville, AL, USA), following the manufacturer’s instructions. Cells were plated in 6-well plates in antibiotic-free media and then transfection was performed with cells at 80% confluence, with a final concentration of 0.5 μg for each vector.

### 2.9. Protein Degradation Assay

Protein degradation assay was used to evaluate the effects of Cul4A on the decay of ANXA10 in lung cancer cells. Cells, transfected with Cul4A siRNA and Cul4A vector, were plated on 6 cm culture dishes. At 80% confluence, the cells were exposed to 100 mg/mL of cycloheximide. Then, the cells were harvested at the indicated time points. Total cellular proteins were extracted and analyzed by western blot analysis using β-actin as a loading control.

### 2.10. Co-Immunoprecipitation Assay

Then, 293T cells were transiently co-transfected with pBabe-Cul4A-myc-his and pCMV6-ANXA10-GFP (OriGene, Rockville, MD, USA) vectors using Lipofectamine 2000 transfection reagent (Invitrogen). Twenty-four hours after transfection, the cells were treated with 10 μg/mL of MG132 (Sigma) for 24 h, and then harvested in a NP-40 lysis buffer (150 mM NaCl, 50 mM Tris [pH 8.0], 1% NP40), protease inhibitor, and phosphatase inhibitor cocktail (Roche, Lewes, UK, USA). Immunoprecipitation was performed using the Catch and Release v2.0 Reversible Immunoprecipitation System (Millipore) according to the manufacturer’s protocols. Anti-GFP (OriGene) and Anti-Cul4A (Abcam) antibodies were used for immunoprecipitation.

### 2.11. In Vivo Ubiquitination Assay

The 293T cells were co-transfected with a combination of pBabe-Cul4A-myc-his and pCMV6-ANXA10-GFP (OriGene) with or without pRK5-HA-Ubiquitin-WT (Addgene, Cambridge, MA, USA). All the cells were treated with 10 µg/mL of MG132 for 24 h prior to lysis. Anti-GFP antibody was used for immunoprecipitation. Anti-HA tag antibody (Cell Signaling, Danvers, MA, USA) was used for the western blot analysis.

### 2.12. Tail Vein Injection Mouse Xenograft Models

Tail vein injection was used to establish a lung metastasis xenograft model for the Cul4A knockdown and metastasis. Approval from the Institutional Animal Care and Use Committee (IACUC) was obtained for the experiments (IACUC No. 2014121206). Female Balb/c athymic nude mice (5–6 weeks old) were housed under specific pathogen-free conditions. Cells were cultured in RPMI media, then suspended at a concentration of 1 × 10^6^ cells/100 μL, and 100 μL of this suspension was injected into the tail vein of the mice. Fluorescence molecular tomography (FMT) (PerkinElmer, Waltham, MA, USA) imaging was performed 6 weeks after injection of the lung cancer cells. ProSense680 (PerkinElmer) was injected into the tail vein and then FMT imaging was performed 48 h later. The mice were euthanized at 8 weeks, and their lungs were harvested for further analysis.

### 2.13. Statistical Analysis

Data are presented as mean values ± standard error of deviation (SD). Student’s *t*-test was used for comparing the means, unless otherwise mentioned. Statistical analysis was carried out using MedCalc version 15 (MedCalc Software, Ostend, Belgium). A *p* value < 0.05 was considered statistically significant. All the statistical tests were two-tailed.

## 3. Results

### 3.1. Cul4A Is Upregulated in the NSCLC Tissues

**Figure 1:**
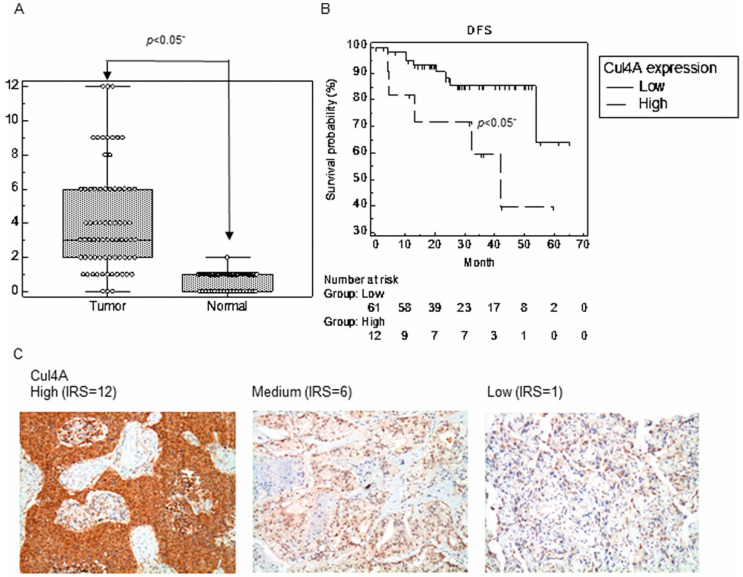

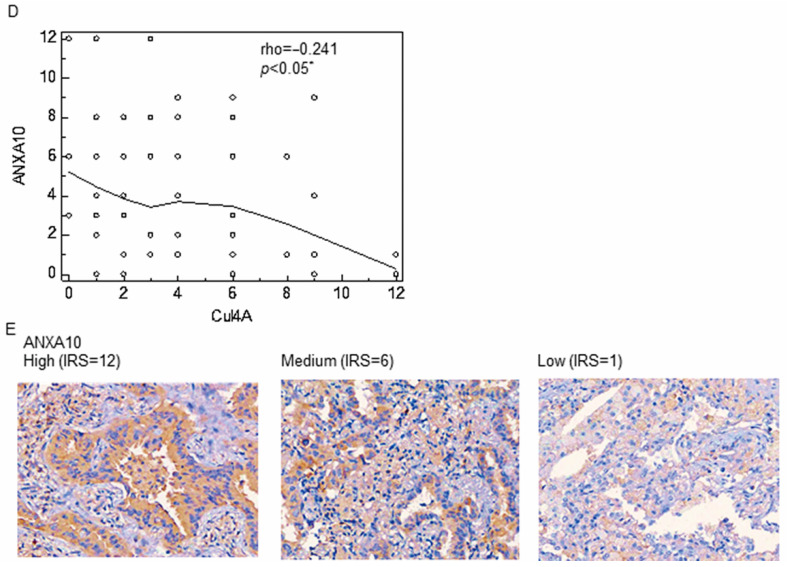
Cul4A is upregulated in non-small cell lung cancer (NSCLC). (**A**) Cul4A levels are determined by IHC staining in the NSCLC tumor and paired normal tissues. The expression of Cul4A is quantified by an IRS score, and the values are expressed as means (±SD). (**B**) Disease-free survival (DFS) of NSCLC patients after surgery is grouped based on a low (IRS ≤ 6) or high (IRS > 6) Cul4A expression. (**C**) Representative images for the expression of Cul4A in NSCLC tissues are shown. Original magnification, 200×. (**D**) Correlation of the expression of Cul4A and ANXA10 in the NSCLC tissues. (**E**) Representative images for the expression of ANXA10 in NSCLC tissues are shown. Original magnification, 200×. IRS: immunoreactive score.

First, we examined the Cul4A protein expression in 73 primary NSCLC tissues. An increased expression of Cul4A was observed in 59 (80.8%) tumor tissues compared to the paired normal tissues (Figure 1A). Receiver operating characteristic (ROC) curves and the Youden index were used to determine the optimal cutoff value of Cul4A IRS for disease recurrence after surgical resection of NSCLC lung cancer (Figure S1). High expression of Cul4A levels, which was defined by an IRS score greater than 6, were detected in 12 of the 73 (16.4%) NSCLC tissue specimens that were analyzed, and the high expression was associated with a significantly decreased disease-free survival (DFS) after surgical resection of the lung cancer (Figure 1B,C). The expression of ANXA10 was also examined and a significantly negative correlation of the expression of Cul4A and ANXA10 was determined using the Spearman rank correlation test (Figure 1D,E).

### 3.2. Knockdown of Cul4A Is Associated with the Upregulation of ANXA10 in Lung Cancer Cells

**Figure 2:**
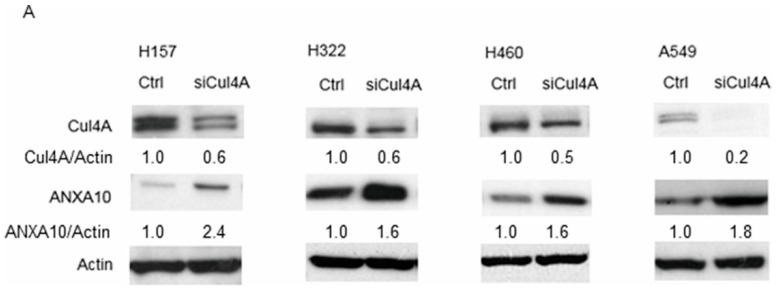

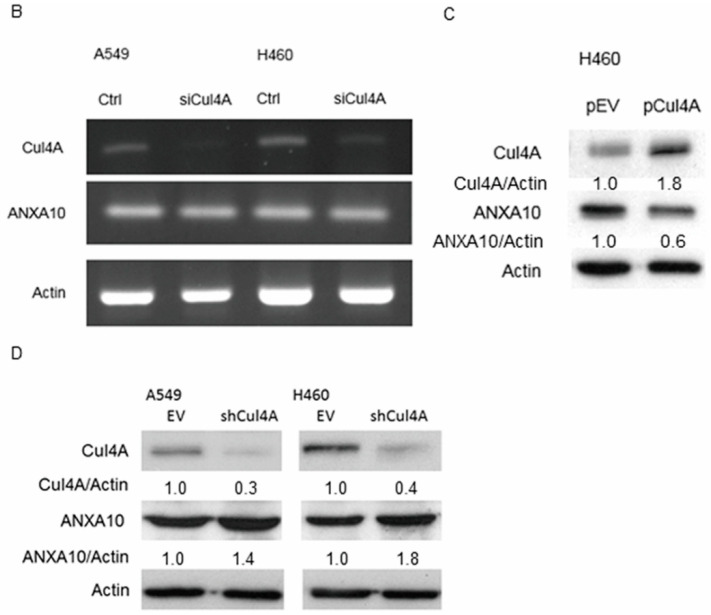
Cul4A knockdown is associated with elevated ANXA10 protein in lung cancer cells. (**A**) Western blot analysis of Cul4A, ANXA10, and actin in H157, H322, H460, and A549 lung cancer cells. Actin was used as the internal control. Ctrl: control siRNA. siCul4A: Cul4A siRNA. (**B**) RT-PCR of *ANXA10* in H460 and A549 lung cancer cells transfected with control and Cul4A siRNA. Actin was used as the internal control. (**C**) Western blot analysis of Cul4A, ANXA10, and actin in H460 lung cancer cells transfected with pcDNA3-Myc3-CUL4A (pCul4A) or empty pcDNA3.1 vector (pEV). (**D**) Western blot analysis of Cul4A, ANXA10, and actin in retroviral Cul4A shRNA (shCul4A) or empty virus (EV) transfected in H460 and A549 lung cancer cells. The expression of Cul4A and ANXA10 was quantified by densitometry and normalized to actin, and using Ctrl, pEV, or EV groups as the control.

We further explored the expression of the cancer metastasis suppressor ANXA10 after knocking down Cul4A. We observed an increase in the ANXA10 protein level in Cul4A knockdown lung cancer cells using western blotting (Figure 2A). The expression of *ANXA10* mRNA was further evaluated by RT-PCR in the Cul4A knockdown H460 and A549 lung cancer cells. Remarkably, no obvious change in the *ANXA10* mRNA levels was observed in the Cul4A shRNA transfected groups of lung cancer cells compared to the cells transfected with the empty vector (Figure 2B). Cul4A overexpression in the H460 lung cancer cells resulted in lowered protein levels of ANXA10 (Figure 2C). Similar to the results observed with transient siRNA transfection, H460 and A549 lung cancer cells were stably transfected with Cul4A shRNA using retroviral transduction, which resulted in the knockdown of Cul4A, and showed increased ANXA10 protein levels (Figure 2D).

### 3.3. Knockdown of Cul4A Represses Metastasis and Invasion in Lung Cancer Cells

**Figure 3:**
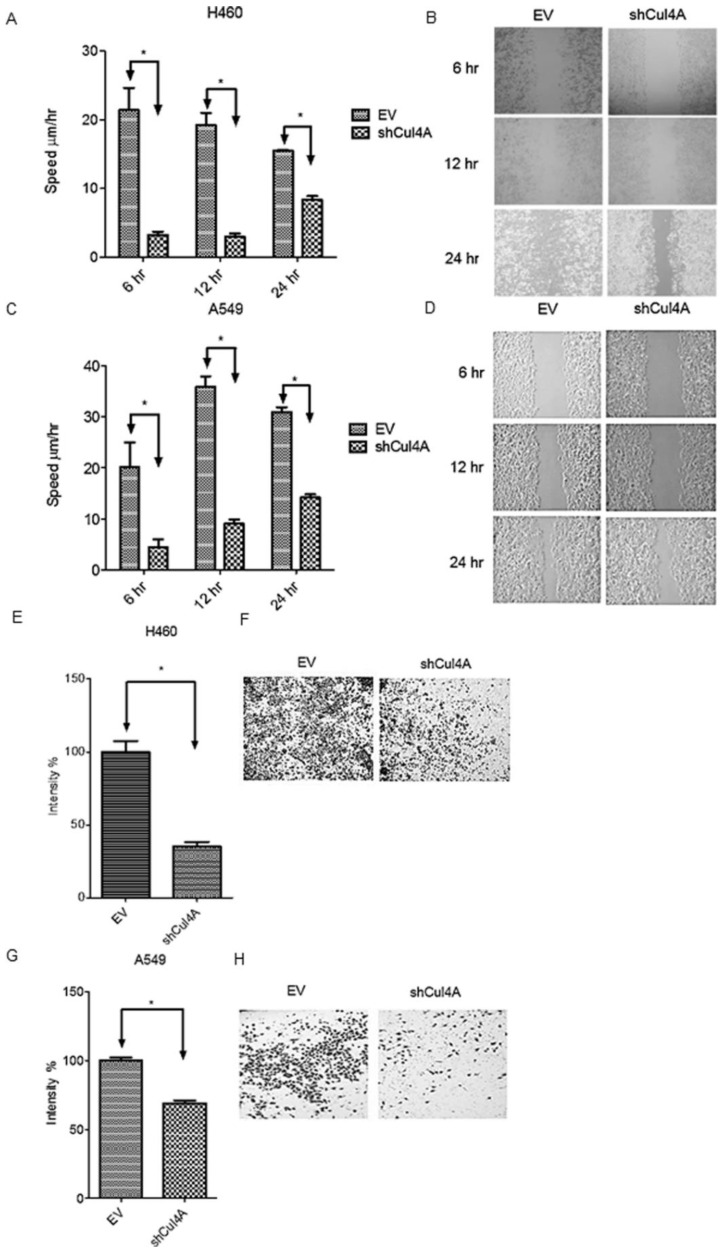
Cul4A knockdown represses metastasis and invasion in lung cancer cells. (**A**,**B**) Cell migration assay of H460 lung cancer cells. (**C**,**D**) Cell migration assay of A549 lung cancer cells. Cell migration was observed at the indicated time points. (**E**,**F**) Cell invasion assay of H460 lung cancer cells. (**G**,**H**) Cell invasion assay of A549 lung cancer cells. Cell invasion was observed after 48 h. H460 and A549 lung cancer cells were transfected with empty vector (EV) and Cul4A shRNA (shCul4A). Experiments were performed in triplicate. Data points represent the average migration speed or cell intensity ± standard deviation. * denotes *p* < 0.05. hr: hours. Original magnification, 100×.

Effects of Cul4A knockdown on metastasis and invasion of Cul4A shRNA transfected H460 and A549 stable lung cancer cells were evaluated using cell migration and invasion assays. We observed that the knockdown of Cul4A significantly repressed metastasis (Figure 3A–D) and invasion (Figure 3E–H) of lung cancer cells.

### 3.4. Cell Migration and Invasion Are Restored by Knockdown of ANXA10 in Cul4A Knockdown Lung Cancer Cells

**Figure 4:**
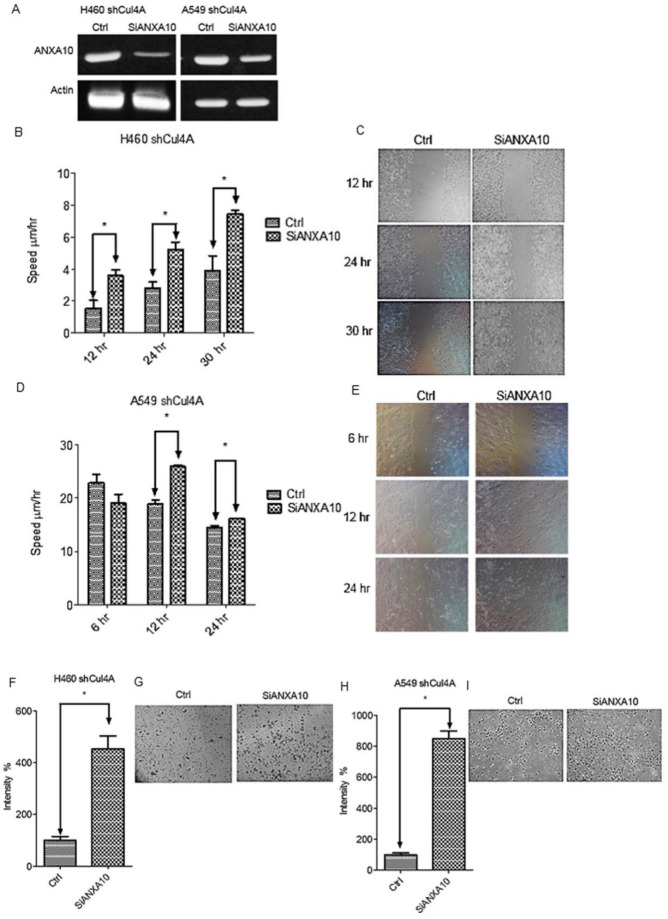
ANXA10 knockdown restores cell migration and invasion in Cul4A knockdown cells. (**A**) RT-PCR of *ANXA10* in H460 (H460 shCul4A) and A549 (A549 shCul4A) Cul4A shRNA transfected stable lung cancer cells or transfected with control siRNA (ctrl) and ANXA10 siRNA (siANXA10). (**B**,**C**) Cell migration assay of H460 shCul4A stable lung cancer cells. (**D**,**E**) Cell migration assay of A549 shCul4A stable lung cancer cells. (**F**,**G**) Cell invasion assay of H460 shCul4A stable lung cancer cells. (**H**,**I**) Cell migration assay of A549 shCul4A stable lung cancer cells. Experiments were performed in triplicate. Data points represent the average migration speed or cell intensity ± standard deviation. * denotes *p* < 0.05. hr: hours.

To evaluate the effect of ANXA10 on the cell migration and invasion of lung cancer cells, ANXA10 knockdown assay using ANXA10 siRNA was performed in Cul4A knockdown H460 and A549 stable lung cancer cells (Figure 4A). Both cell migration (Figure 4B–E) and invasion (Figure 4F–I) were restored following the knockdown of ANXA10 in Cul4A knockdown H460 and A549 lung cancer stable cells.

### 3.5. Knockdown of Cul4A Represses Metastasis of Lung Cancer Tumors in Tail Vein Injection Mouse Models

**Figure 5:**
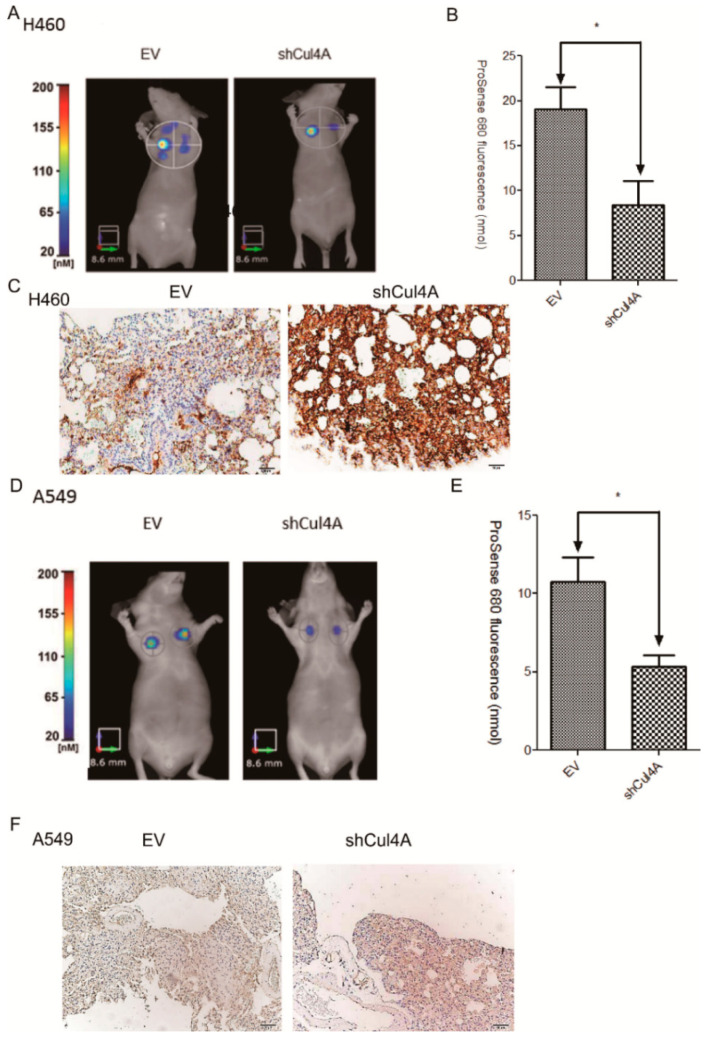
Cul4A knockdown represses metastasis of lung cancer tumors in tail vein injection mouse models. (**A**,**B**) Fluorescence molecular tomography (FMT) imaging of the H460 lung cancer stable cells transfected with empty virus (EV) or Cul4A shRNA (shCul4A) retrovirus in a tail vein injection model, 6 weeks after injection of the lung cancer cells. ProSense680 was injected into the tail vein, and FMT imaging was performed 48 h later. (**C**) IHC of ANXA10 in the H460 EV and shCul4A lung tumors. (**D**,**E**) FMT imaging of the A549 lung cancer stable cells transfected with empty virus (EV) or Cul4A shRNA (shCul4A) retrovirus in a tail vein injection model, 6 weeks after injection of the lung cancer cells. ProSense680 was injected into the tail vein, and FMT imaging was performed 48 h later. (**F**) IHC of ANXA10 in the A549 EV and shCul4A lung tumors. Data points represent the average of fluorescence ± standard deviation. * denotes *p* < 0.05. Scale bar: 50 μm.

Tail vein injection models were established using H460 and A549 cells that were stably transfected with Cul4A shRNA to confirm the effect of Cul4A on lung cancer metastasis. A significant reduction in lung metastasis was observed in the Cul4A knockdown groups of H460 using FMT imaging (Figure 5A,B) and A549 (Figure 5D,E) lung cancer cells.

An increased expression of ANXA10 was also observed in the Cul4A knockdown H460 (Figure 5C) and A549 cells compared to the empty virus transfected lung cancer cells (Figure 5F).

### 3.6. ANXA10 Expression Is Regulated by Protein Degradation

**Figure 6:**
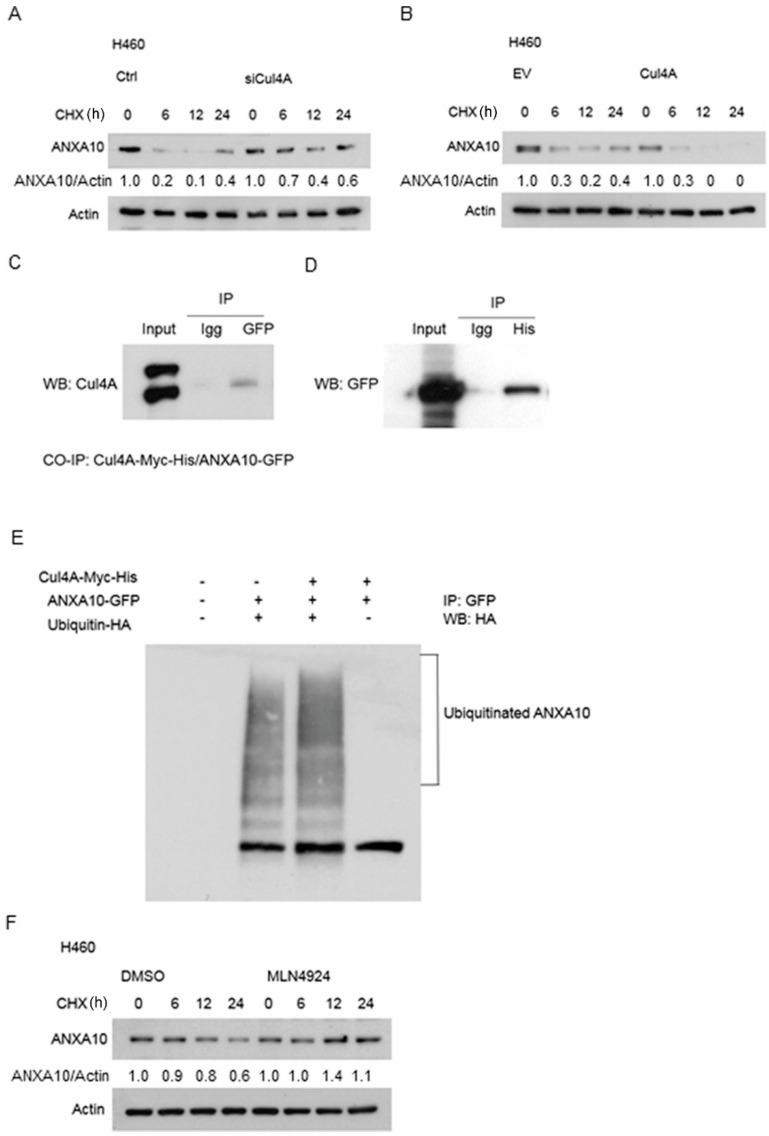
ANXA10 is ubiquitinated by Cul4A through a protein–protein interaction stimulating degradation. (**A**) Protein degradation assay for ANXA10 in the H460 lung cancer cells. H460 lung cancer cells were transfected with Cul4A siRNA (siCul4A) or control siRNA (ctrl). (**B**) Protein degradation assay for ANXA10 in the H460 lung cancer cells. H460 lung cancer cells were transfected and overexpressed with pcDNA3-Myc3-CUL4A (Cul4A) or empty pcDNA3.1 vector (EV). H460 cells were incubated with 100 µg/mL cycloheximide (CHX) for the indicated time periods (h: hour). The expression of ANXA10 was quantified by densitometry and normalized to actin, and using o hour groups as the control. (**C**,**D**) Reciprocal immunoprecipitation of Cul4A and ANXA10. The pBabe-Cul4A-myc-his and pCMV6-ANXA10-GFP vectors were co-transfected into 293T cells. Anti-GFP and anti-His tag antibodies were used for the immunoprecipitation. (**E**) In vivo ubiquitination of ANXA10 and Cul4A. The pCMV6-ANXA10-GFP (ANXA10-GFP), pBabe-Cul4A-myc-his (Cul4A-Myc-His), and pRK5-HA-Ubiquitin-WT (Ubiquitin-HA) vectors were transfected into the 293T cells. In reciprocal immunoprecipitation and in vivo ubiquitination assays, the cells were treated with 10 µg/mL of MG132 for 24 h prior to being lysed. Anti-GFP antibody was used for the immunoprecipitation. Anti-HA antibody was used for the western blot (WB) analysis. (**F**) Protein degradation assay for ANXA10 in H460 lung cancer cells treated with DMSO and 1 μM of MLN4924, a NEDD8 inhibitor, for 24 h. H460 cells were incubated with 100 µg/mL cycloheximide (CHX) for the indicated time periods. The expression of ANXA10 was quantified by densitometry normalized to actin, and using o hour groups as the control.

Protein degradation assay was used to evaluate the stability of ANXA10 protein in Cul4A knockdown and overexpressed H460 lung cancer cells. After treated with cycloheximide for the indicated periods, protein degradation was dramatically increased in the Cul4A overexpressed lung cancer cells (Figure 6A), while Cul4A knockdown lung cancer cells showed a marked reduction in protein degradation (Figure 6B). The NEDD8 inhibitor specifically blocks the NEDDylation and subsequent function of Cul4A [30]. Protein degradation assay was also performed in H460 lung cancer cells after the treatment of MLN4924 (Sigma, St. Louis, MO, USA), a NEDD8 inhibitor. Decreased protein degradation was observed in the MLN4924 treated lung cancer cells (Figure 6F).

### 3.7. ANXA10 Is Ubiquitinated by Cul4A Through a Protein–Protein Interaction

We evaluated the role of Cul4A mediated ubiquitination in ANXA10 degradation. Co-immunoprecipitation assay was performed and the association of ANXA10 with Cul4A was observed (Figure 6C,D). Further in vivo ubiquitination assays showed that the overexpression of Cul4A increased the ubiquitination of ANXA10 in the 293T cells (Figure 6E).

## 4. Discussion

Cul4A has been implicated in multiple cancers, namely breast [7], mesothelioma [8], lung [9], and liver cancers [10]. In this study, we observed that the upregulated Cul4A is associated with poor prognosis in NSCLC lung cancer patients after surgery. On the other hand, the knockdown of Cul4A is associated with the decreased invasion and metastasis of lung cancer cells and tumors. The knockdown of Cul4A is associated with the increased expression of ANXA10, a tumor suppressor protein, in lung cancer cells. Further experimentation revealed that Cul4A regulates ANXA10 through ubiquitination and protein degradation in lung cancer cells. We hypothesized that Cul4A mediated degradation of ANXA10 was one of the key mechanisms in lung cancer invasion and metastasis. To our knowledge, the association of Cul4A and ANXA10 in NSCLC tissues and lung cancer cells was also reported for the first time after reviewing published papers.

A nude mouse tail vein injection metastasis model was established in our study, which showed a decreased metastatic potential in Cul4A knockdown lung cancer cells. Cul4A has been reported to promote cancer metastasis and invasion in osteosarcoma [31] cells. In breast cancer cells, Cul4A induces an epithelial–mesenchymal transition, and it promotes cancer metastasis by regulating the expression of the EMT regulatory ZEB1 gene [32]. In colorectal cancers, overexpression of Cul4A induces the epithelial–mesenchymal transition through the regulation of H3K4 trimethylation at the E-cadherin, N-cadherin, and vimentin gene promoters [33]. In gastric cancers, overexpression of Cul4A promoted gastric cancer cell proliferation and epithelial–mesenchymal transition by downregulating LATS1-Hippo-YAP signaling [34]. Our study showed that the overexpression of Cul4A was associated with the downregulation of the tumor suppressor ANXA10 in lung cancer tissues and cells, which may provide another mechanism for the role of Cul4A in lung cancer invasion and metastasis. The mechanism of lung cancer invasion and metastasis regulated by Cul4A is complex, and further studies regarding Cul4A and other metastasis suppressors are still warranted in the future.

ANXA10 is the latest identified member of the annexin family of calcium (Ca^2+^) and phospholipid-binding proteins [35]. The downregulation of ANXA10 correlates with decreased differentiation, invasion, and tumor progression, pointing to a possible tumor suppressor role [35]. In bladder cancer, the downregulation of ANXA10 is also related to the aggressiveness of the cancer [25]. In hepatocellular carcinoma, the downregulation of ANXA10 correlates with p53 mutation and it is associated with vascular invasion, tumor progression, and poor prognosis [36]. Decreased ANXA10 has been correlated with increased invasion in a colorectal cancer cell line and with the increased proliferation and migration in a gastric cancer cell line [37]. Additionally, the upregulation of S100A4, which is considered a mediator of metastasis, has been reported to downregulate ANXA10 in a lung cancer cell line [38]. In our study, the knockdown of ANXA10 increases lung cancer cells migration and invasion. These reports together show strong evidence of the tumor suppressor and metastasis role of ANXA10 in cancer cells.

In conclusion, our results showed that Cul4A is important in lung cancer cell invasion and metastasis through the inhibition of ANXA10, a tumor suppressor. Our results add ANXA10 to the repertoire of tumor suppressor proteins that are inhibited by Cul4A in cancer, and thus, it suggests Cul4A as a potential drug target for the development of novel therapy for lung cancer in the future.

## 5. Conclusions

Our findings suggest that Cul4A is a prognostic marker in NSCLC patients after surgery of lung cancer. Cul4A also plays important roles in lung cancer invasion and metastasis partially through ubiquitin-mediated protein degradation of ANXA10 in lung cancer cells. The role of ANXA10 as a tumor and metastasis suppressor in lung cancer cells was further confirmed.

## Supplementary Materials

The following are available online at https://www.mdpi.com/2072-6694/11/5/618/s1, Figure S1: ROC curve of the Cul4A IRS.
