# Correction: Hung et al. Cul4A Modulates Invasion and Metastasis of Lung Cancer through Regulation of ANXA10. *Cancers* 2019, *11*, 618

**DOI:** 10.3390/cancers17142377

**Published:** 2025-07-17

**Authors:** Ming-Szu Hung, Yi-Chuan Chen, Paul-Yann Lin, Ya-Chin Li, Chia-Chen Hsu, Jr-Hau Lung, Liang You, Zhidong Xu, Jian-Hua Mao, David M. Jablons, Cheng-Ta Yang

**Affiliations:** 1 Division of Thoracic Oncology, Department of Pulmonary and Critical Care Medicine, Chang Gung Memorial Hospital, Chiayi branch 61363, Taiwan; c39958@yahoo.com.tw; 2 Department of Medicine, College of Medicine, Chang Gung University, Taoyuan 33302, Taiwan; 3 Department of Respiratory Care, Chang Gung University of Science and Technology, Chiayi Campus, Chiayi 61363, Taiwan; 4 Department of Emergency Medicine, Chang Gung Memorial Hospital, Chiayi branch 61363, Taiwan; giomacky@gmail.com; 5 Department of Anatomic Pathology, Dalin Tzu Chi Hospital, Buddhist Tzu Chi Medical Foundation, Chiayi 62247, Taiwan; linpauly0018@gmail.com; 6 Department of Hematology and Oncology, Chang Gung Memorial Hospital, Chiayi branch 61363, Taiwan; loofahhsu@gmail.com; 7 Department of Medical Research and Development, Chang Gung Memorial Hospital, Chiayi branch 61363, Taiwan; jrhaulung@gmail.com; 8 Thoracic Oncology Laboratory, Department of Surgery, Comprehensive Cancer Center, University of California, San Francisco, CA 94143, USA; liang.you@ucsf.edu (L.Y.); zhidong.xu@gmail.com (Z.X.); david.jablons@ucsfmedctr.org (D.M.J.); 9 Life Sciences Division, Lawrence Berkeley National Laboratory, One Cyclotron Road, Berkeley, CA 94720, USA; jhmao@lbl.gov; 10 Department of Respiratory Care, College of Medicine, Chang Gung University, Taoyuan 33302, Taiwan; yang1946@cgmh.org.tw; 11 Department of Pulmonary and Critical Care Medicine, Chang Gung Memorial Hospital, Taoyuan branch 33378, Taiwan

## Error in Figure

In the original publication [1], there was a mistake in Figures 3B and 4E as published. During the figure preparation for the submission, the image for EV 6 h was mistakenly duplicated as shCul4A 6 h in Figure 3B. Similarly, the image for Ctrl 6 h was inadvertently used in place of SiANXA10 6 h in Figure 4E. These errors were corrected by replacing the duplicated panels with the appropriate images for shCul4A 6 h in Figure 3B and 37 SiANXA10 6 h in Figure 4E. The corrected Figure 3B and Figure 4E appear below. The authors apologize for any inconvenience caused and state that the scientific conclusions are unaffected. This correction was approved by the Academic Editor. The original publication has also been updated.

## Figures and Tables

**Figure 3:**
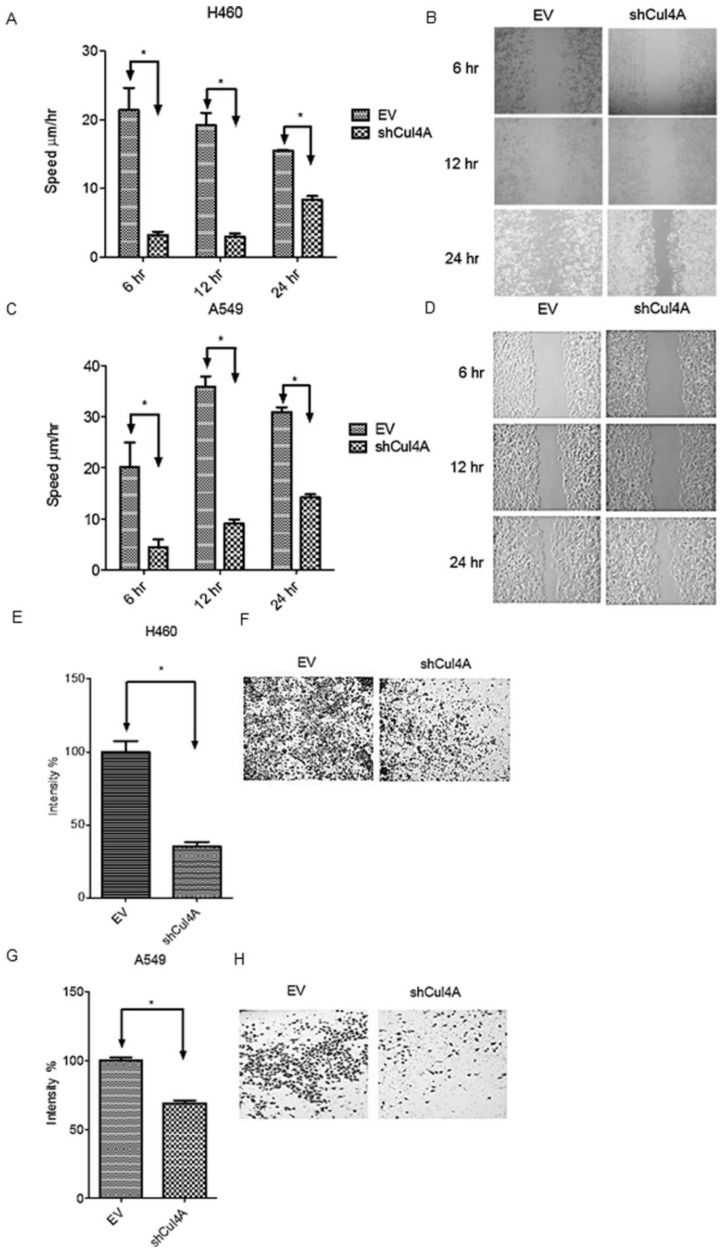
Cul4A knockdown represses metastasis and invasion in lung cancer cells. (**A**,**B**) Cell migration assay of H460 lung cancer cells. (**C**,**D**) Cell migration assay of A549 lung cancer cells. Cell migration was observed at the indicated time points. (**E**,**F**) Cell invasion assay of H460 lung cancer cells. (**G**,**H**) Cell invasion assay of A549 lung cancer cells. Cell invasion was observed after 48 h. H460 and A549 lung cancer cells were transfected with empty vector (EV) and Cul4A shRNA (shCul4A). Experiments were performed in triplicate. Data points represent the average migration speed or cell intensity ± standard deviation. * denotes *p* < 0.05. hr: hours. Original magnification, 100×.

**Figure 4:**
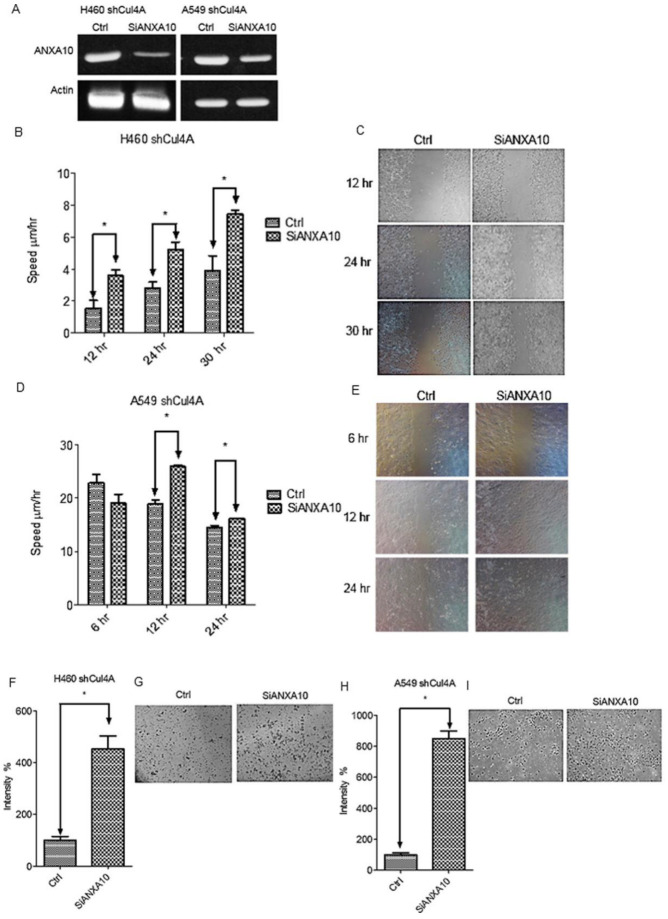
ANXA10 knockdown restores cell migration and invasion in Cul4A knockdown cells. (**A**) RT-PCR of ANXA10 in H460 (H460 shCul4A) and A549 (A549 shCul4A) Cul4A shRNA transfected stable lung cancer cells or transfected with control siRNA (ctrl) and ANXA10 siRNA (siANXA10). (**B**,**C**) Cell migration assay of H460 shCul4A stable lung cancer cells. (**D**,**E**) Cell migration assay of A549 shCul4A stable lung cancer cells. (**F**,**G**) Cell invasion assay of H460 shCul4A stable lung cancer cells. (**H**,**I**) Cell migration assay of A549 shCul4A stable lung cancer cells. Experiments were performed in triplicate. Data points represent the average migration speed or cell intensity ± standard deviation. * denotes *p* < 0.05. hr: hours.
